# Genome Sequence of Vibrio natriegens Phage vB_VnaS-AQKL99

**DOI:** 10.1128/MRA.00967-20

**Published:** 2020-10-01

**Authors:** Noah Yonas, Paige Boleman, Y Nguyen, Makenzie Kerr, Kema Malki, Anthony M. Greco, Mya Breitbart

**Affiliations:** a College of Marine Science, University of South Florida, Saint Petersburg, Florida, USA; DOE Joint Genome Institute

## Abstract

Vibrio natriegens is a naturally occurring marine bacterium that is emerging as a microbiological model system. Here, we describe Aquatic Killer 99 (AQKL99), a novel phage that infects Vibrio natriegens 14048. The genome of the phage is 58,464 bp long, has a GC content of 45.9%, and contains 51 protein-coding genes.

## ANNOUNCEMENT

*Vibrio* spp. are Gram-negative proteobacteria that inhabit saline and estuarine environments; however, several species are human pathogens (1). The characterization of phages infecting *Vibrio* spp. is of broad interest for the fields of ecology, evolution, human health, and biotechnology (2). Vibrio natriegens is emerging as a microbiological model system due to its rapid doubling time (<10 min) under standard laboratory conditions and the availability of tools for genetic manipulation (3, 4). Here, we describe the isolation and genome annotation of Aquatic Killer 99 (AQKL99), a novel phage that infects Vibrio natriegens 14048.

**FIG 1:**
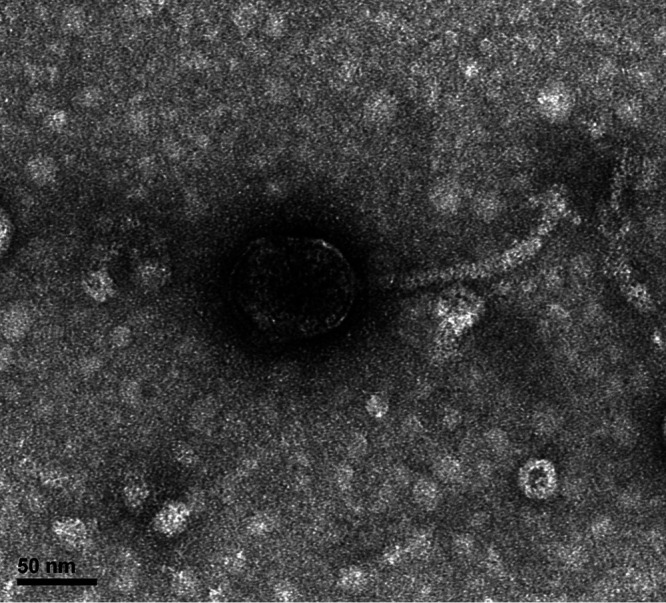
Transmission electron micrograph of *Vibrio* phage vB_VnaS-AQKL99.

In June 2019, 3 liters of surface seawater from Bayboro Harbor (Tampa Bay, FL, USA) was prefiltered through a 0.8-μm filter, and viruses were concentrated using iron chloride flocculation (5). A double-layer plaque assay on Vibrio natriegens 14048 on tryptic soy agar with 10 g per L sodium chloride at 25°C led to the isolation of a novel phage, which was subsequently plaque purified three times (6). Phage lysates were adsorbed to a copper grid, negatively stained with 2% (wt/vol) uranyl acetate (7), and viewed on a Hitachi 7100 transmission electron microscope equipped with a Gatan Orius high-resolution digital camera. The morphology of phage AQKL99 is consistent with that of members of the family *Siphoviridae*, with an average capsid diameter of 74 ± 18 nm and a long, flexible tail averaging 131 ± 38 nm, based on pixel measurements relative to the scale bar of 40 phage particles (Fig. 1). Phage AQKL99 was unable to produce plaques on 53 *Vibrio* colonies isolated from the same seawater sample on thiosulfate-citrate-bile salts-sucrose (TCBS) agar, suggesting a narrow host range. However, neither the identity nor the uniqueness of these *Vibrio* isolates was determined.

Phage DNA was isolated from a 0.2-μm-filtered overnight lysate with the Qiagen MinElute virus spin kit and was sent to the Microbial Genome Sequencing (MiGS) Center for library preparation with the Illumina Nextera kit and sequencing on the NextSeq 550 platform. Sequences were processed using FastQC v0.11.5 (8) with default parameters, assembled using SPAdes v3.11.1 (9), and evaluated using QUAST v5.0.1 (10) to reveal a single contig with ∼2,900× coverage. This contig, which was presumed to represent the complete linear genome of phage AQKL99, is 58,464 bp long with a GC content of 45.9%. As determined with Geneious Prime v2020.1.2 (11), phage AQKL99 shares 75% genome-wide pairwise identity with *Vibrio* phage VhaS-tm (GenBank accession number KX198614), which was isolated from oyster tissue and can prevent Vibrio harveyi from causing disease in greenlip abalone (*Haliotis laevigata*) (12).

Open reading frames (ORFs) were predicted through Geneious Prime v2020.1.2 using default parameters, which resulted in the identification and annotation of 51 protein-coding genes, mostly transcribed in the reverse direction (11). Amino acid sequence similarity searches were performed against the nonredundant database using BLASTp with default parameters and an E value cutoff value of <0.001 (13). Approximately 75% of the genes are of unknown function; however, the phage AQKL99 genome encodes several putative structural proteins (major capsid protein, minor head protein, portal protein, tail assembly protein, tail length tape measure protein, and large and small terminase subunits), DNA replication proteins (DNA helicase, DNA polymerase, DNA replicative clamp, and primase), and other notable proteins (autolysin *N*-acetylmuramoyl-l-alanine amidase, AAA family ATPase, and multimodular transpeptidase-transglycosylase). Interestingly, the phage AQKL99 genome encodes three proteins putatively involved in deazaguanine DNA modification (QueC, QueD, and QueD), which is a common nucleotide substitution used to protect phage DNA from host restriction enzymes (14).

### Data availability.

The genome sequence and associated metadata for *Vibrio* phage vB_VnaS-AQKL99 are available in GenBank under the accession number MT795651.1. Raw reads are available in the SRA database under the accession number SRX9058260.
